# Supplementary material for: Multiple acyl-CoA dehydrogenase deficiency kills Mycobacterium tuberculosis in vitro and during infection
Source: Nat Commun. 2021 Nov 15;12:6593. doi: 10.1038/s41467-021-26941-1 (PMC8593149; doi:10.1038/s41467-021-26941-1)
Supplement: Supplementary file 1 — Supplementary Information [file 41467_2021_26941_MOESM1_ESM.pdf]

Supplementary Information

Multiple acyl-CoA dehydrogenase deficiency kills *Mycobacterium tuberculosis* in vitro and during infection

Tiago Beites, Robert S Jansen, Ruojun Wang, Adrian Jinich, Kyu Y Rhee, Dirk Schnappinger, Sabine Ehrt

Supplementary Figures 1-8

Supplementary Tables 1-4

Supplementary References 1-3

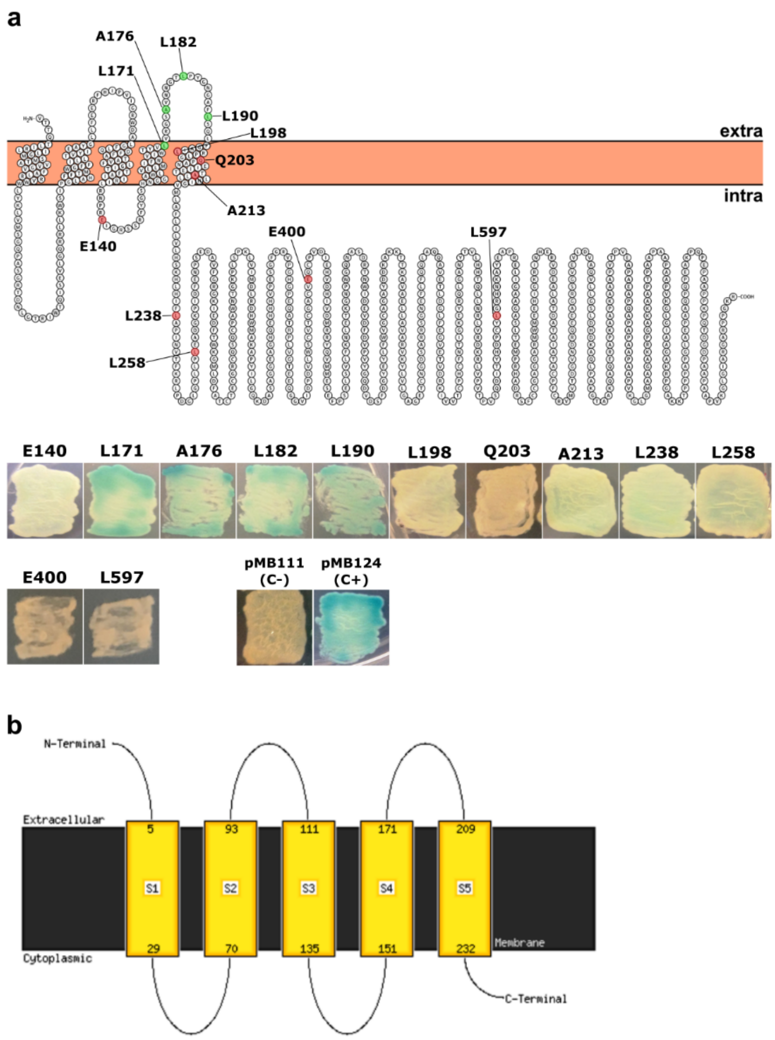

Supplementary Fig. 1 EtfD has 5 transmembrane domains and the soluble portion faces the periplasm. **a**, *E. coli* alkaline phosphatase PhoA was fused at different residues of the EtfD (Rv0338c) transmembrane domain and soluble portion. The constructs were expressed in *Mycobacterium smegmatis* and the strains grew in the presence of the chromogenic PhoA substrate BCIP. As positive control (c+) PhoA is fused with the antigen 85B that contains a secretion signal (PhoA requires the oxidative environment of the periplasm to be active), while the negative control (c-) lacks any secretion signal. These results are representative of 2 independent experiments. **b**, EtfD (Rv0338c) topology prediction with the MEMSAT algorithm.

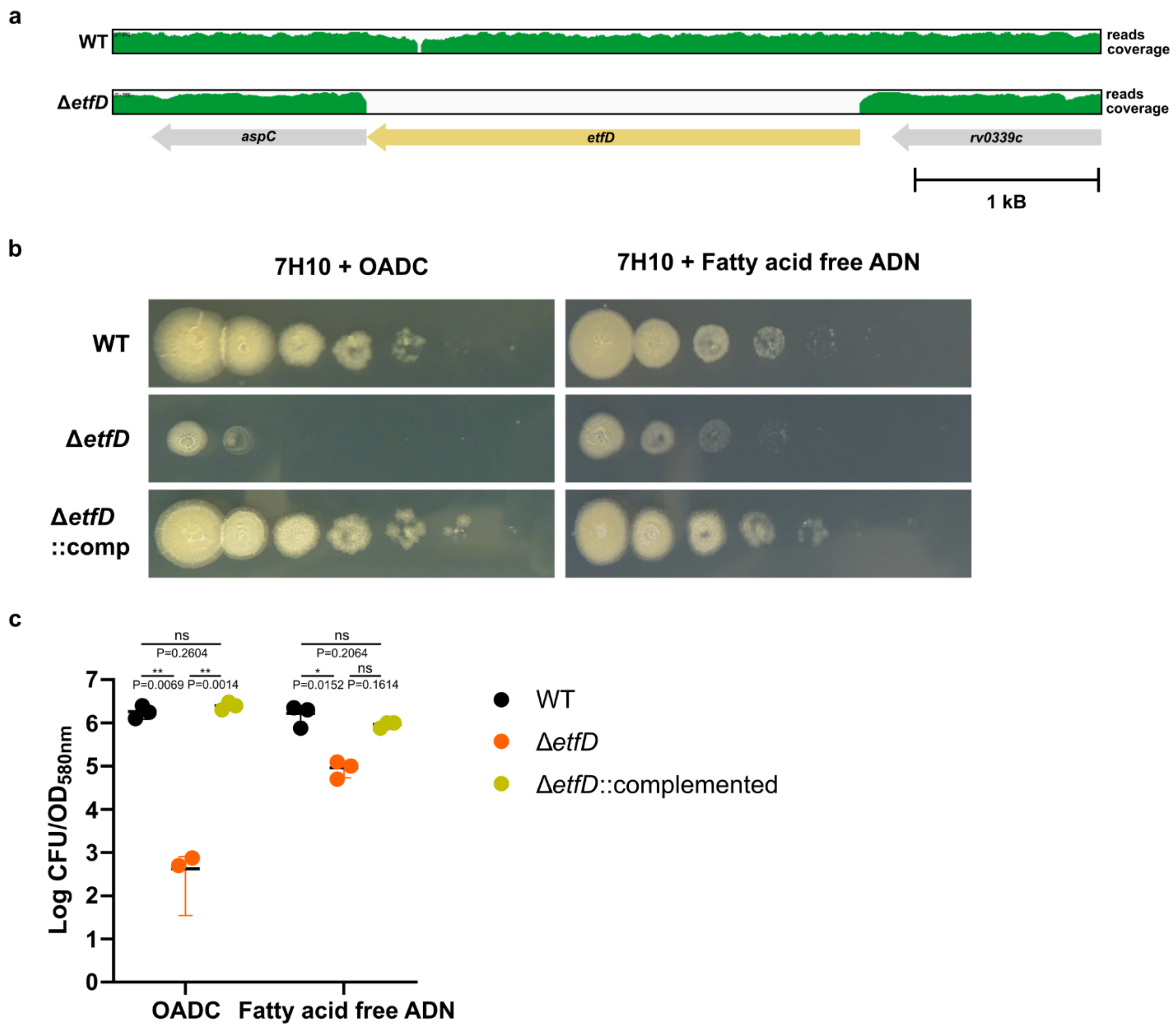

Supplementary Fig. 2. Δ*etfD* genetic identity confirmation. **a**, Whole genome sequencing coverage showing the *etfD* wild-type locus and gene deletion in Δ*etfD.* **b**, Spot assay on solid media. Serial dilutions (10^6 down to 1 bacteria) were inoculated in 7H10 supplemented with OADC or fatty acid free ADN and incubated for 14 days. The spot assays are representative of 3 independent experiments. **c**, Quantification of spot assays presented in (**b)** are aggregates of the data from the 3 independent experiments. To quantify the spot assays, we have picked the first dilution where isolated CFUs could be counted and divided it by the optical density (OD) of the corresponding bacterial suspension. The discrepancy between the OD of the bacterial suspension and the corresponding CFUs, which is due to sensitivity to lipids in the solid media, is reflected by this ratio. Data are averages with individual data points depicted. Error bars correspond to standard deviation. Statistical significance was assessed by one-way ANOVA followed by post hoc test (Tukey test; GraphPad Prism). *$P < 0.05$; **$P < 0.01$; ***$P < 0.001$. ns – not significant.

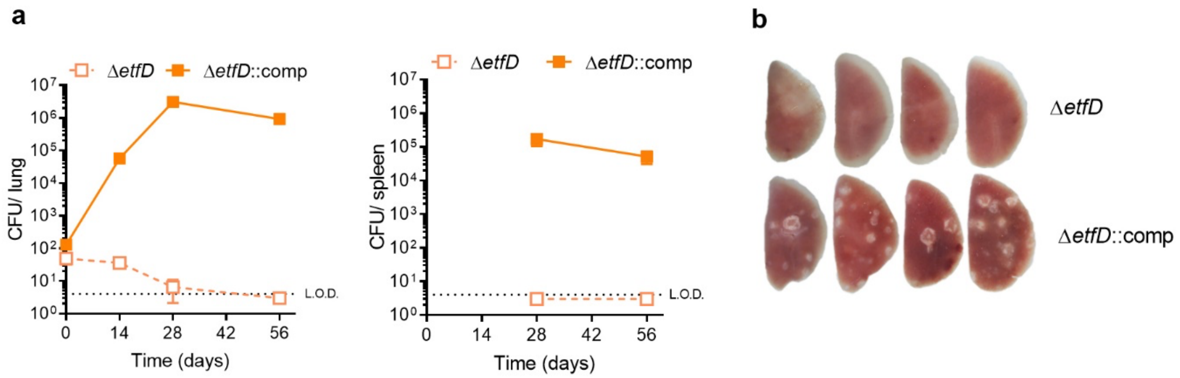

Supplementary Fig. 3. EtfD is essential for growth and survival in vivo. Second experiment of growth and persistence of Δ*etfD* in vivo. **a**, Growth and persistence of Δ*etfD* and the complemented mutant in mouse lungs and spleens. Data are CFU averages from four mice per time point and are representative of two independent experiments. Error bars correspond to standard deviation. “Comp” stands for complemented. L.O.D. stands for limit of detection. **b**, Gross pathology of lungs infected with Δ*etfD* and the complemented mutant at day 56.

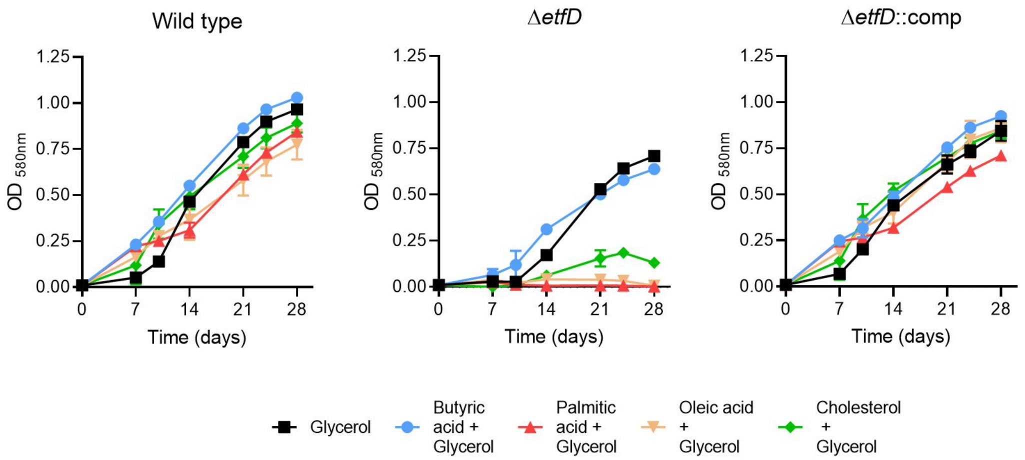

Supplementary Fig. 4. Long-chain fatty acids are toxic to Δ*etfD*. Strains were grown in media with mixed carbon sources containing 25 mM glycerol and 250 μM cholesterol or the following fatty acids: 2.5 mM butyric acid, 250 μM palmitic acid and 250 μM oleic acid. Glycerol (25 mM) alone was used as a positive control for Δ*etfD*. Palmitic acid and oleic acid were replenished every 3 to 4 days to sustain growth and avoid toxicity for the first 14 days. Cholesterol was replenished every to 3 to 4 days for the first 14 days to minimize precipitation. Data are averages of 3 replicates and are representative of 3 independent experiments. Error bars correspond to standard deviation. "Comp" stands for complemented.

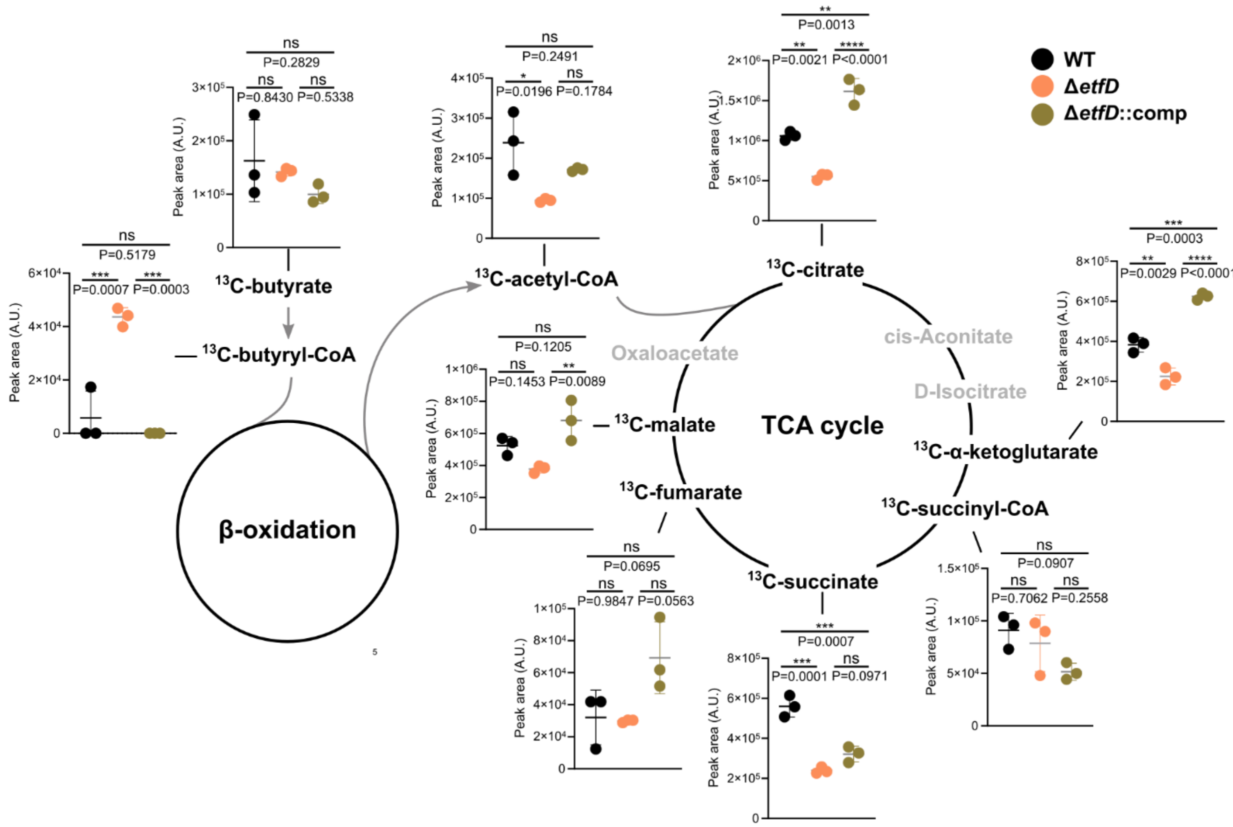

Supplementary Fig. 5. Stable isotope tracing reveals a block in β-oxidation at the level of acyl-coA dehydrogenases. Strains were grown on filters on top of solid medium permissible to Δ*etfD* growth for 7 days and then transferred to solid media with $^{13}C_4$-labelled butyric acid (2.5 mM) as single carbon source for 24 hrs. Levels of the indicated ^{13}C-labeled metabolites (total ^{13}C pool, except for $^{13}C_4$-butyrate) were quantified by LC-MS analysis. Data are averages of 3 replicates of the second independent experiment. Error bars correspond to standard deviation. “Comp” stands for complemented. Statistical significance was assessed by one-way ANOVA followed by post hoc test (Tukey test; GraphPad Prism). *P < 0.05; **P < 0.01; ***P < 0.001; ****P < 0.0001. ns – not significant.

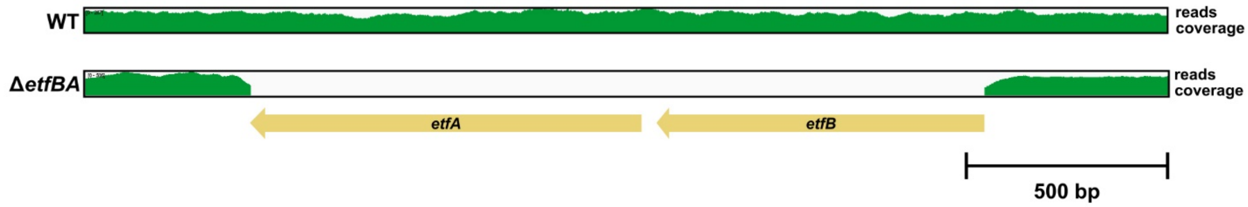

Supplementary Fig. 6. Δ*etfBA* genetic identity confirmation. Whole genome sequencing coverage showing the *etfBA* wild-type loci and gene deletion in Δ*etfBA*.

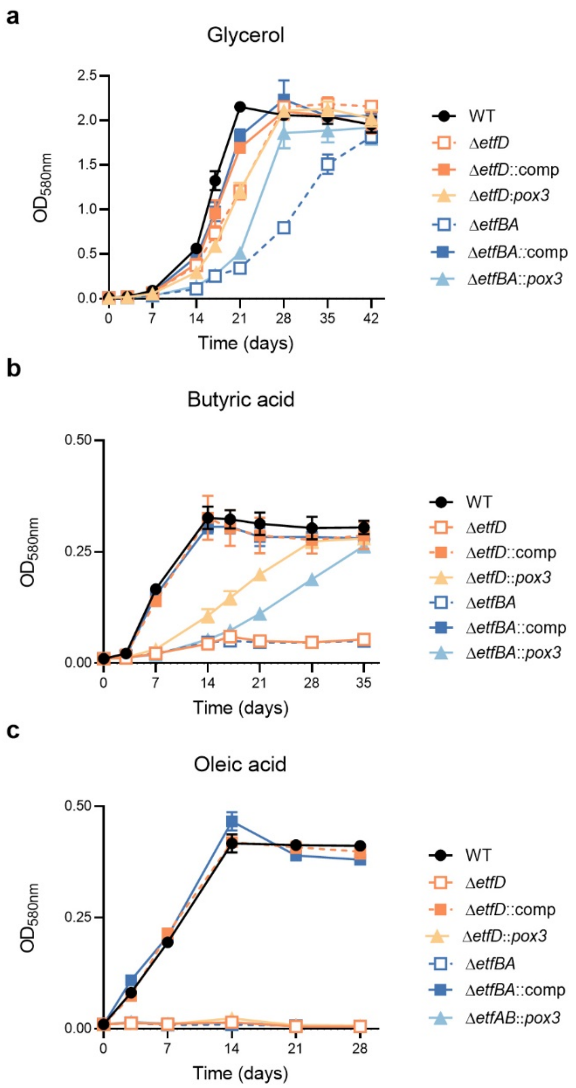

Supplementary Fig. 7. Growth of Δ*etfD* and Δ*etfBA* expressing the acyl-CoA oxidase Pox3 in different carbon sources. Strains were grown in media with 25 mM glycerol (**a**), 2.5 mM butyric acid **(b**), and 250 μM oleic acid (**c**). Oleic acid was replenished every 3 to 4 days to sustain growth and avoid toxicity. Data are averages of 3 replicates and are representative of 3 independent experiments. Error bars correspond to standard deviation. "Comp" stands for complemented.

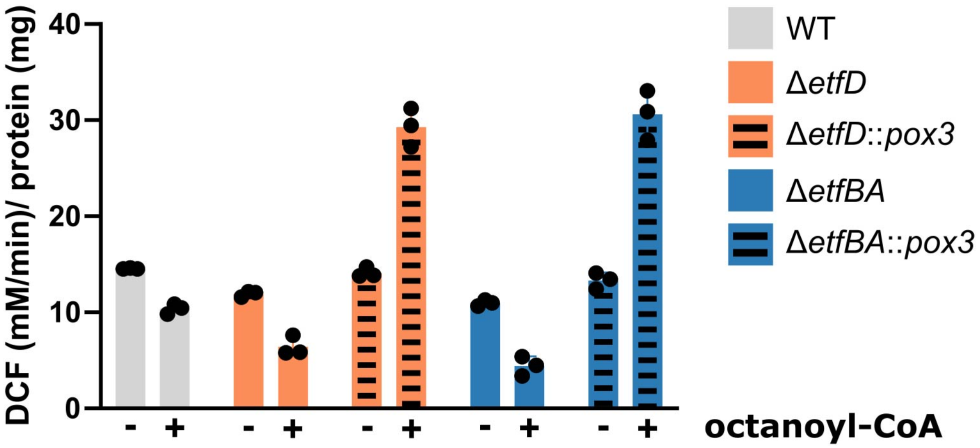

Supplementary Fig. 8. In vitro acyl-CoA oxidase activity in cell lysates. To detect specific Pox3 activity we performed acyl-CoA activity assays with and without the substrate octanoyl-CoA. The basal activity observed in all the strains is likely due to spontaneous and unspecific generation of hydrogen peroxide. Data are averages of 3 replicates and are representative of 3 independent experiments. Error bars correspond to standard deviation.

Supplementary Table 1. ***Mycobacterium tuberculosis*** **whole genome sequencing analysis of Δ*etfD* and Δ*etfBA*.** This table presents the genetic polymorphisms present in the knockout strains in comparison with the parental strain.

STRAIN	POSITION	GENE	DESCRIPTION	AA CHANGE	REF	ALT	# REF READS	# ALT READS	COVERAGE
Δ*etfD*	132415	*rv0109*	PE_PGRS1	R346G	C	G	1	411	412
	210094	*rv0179c*	lprO	silent	A	G	2	515	517
	528357	NA	intergenic bet Rv0439c and Rv0440	NA	C	A	354	406	760
	837036	*rv0746*	PE_PGRS9	T445A	A	G	0	406	406
	986207	NA	intergenic bet Rv0887c and Rv0888	NA	C	A	0	531	531
	2874461	*rv2553c*	Rv2553c	inframe ins V58	G	GCCA	5	256	261
	2933205	*rv2604c*	snoP	D151E	G	T	0	482	482
	3121725	NA	intergenic bet Rv2813 and Rv2816c	NA	GGGTTTCCGTCCCCT CTCGGGGTTTTGGGT CTGACGACATGCTGA GCTGAGGCGCCGGAT GATGGTGGTGCTGAA	G	15	193	208
	3961169	*rv3523*	ltp3	silent	G	C	0	445	445
Δ*etfBA*	132415	*rv0109*	PE_PGRS1	R346G	C	G	0	342	342
	210094	*rv0179c*	lprO	silent	A	G	3	368	371
	837036	*rv0746*	PE_PGRS9	T445A	A	G	0	307	307
	986207	NA	intergenic bet Rv0887c and Rv0888	NA	C	A	0	443	443
	2874461	*rv2553c*	Rv2553c	inframe ins V58	G	GCCA	29	191	220
	2933205	*rv2604c*	snoP	D151E	G	T	0	326	326
	3121725	NA	intergenic bet Rv2813 and Rv2816c	NA	GGGTTTCCGTCCCCT CTCGGGGTTTTGGGT CTGACGACATGCTGA GCTGAGGCGCCGGAT GATGGTGGTGCTGAA	G	11	114	125
	4085080	*rv3645*	Rv3645	D307E	C	A	5	398	403

Ref - Reference sequence

Alt - Alternative sequence

Supplementary Table 2. EtfD interactors identified by mass spectrometry.

Rv	Gene	Description	EtfD-Flag : Control ratio
Rv0016c	pbpA	Probable penicillin-binding protein PbpA	13.10
Rv0050	ponA1	Probable bifunctional penicillin-binding protein 1A/1B PonA1 (murein polymerase) (PBP1): penicillin-insensitive transglycosylase (peptidoglycan TGASE) + penicillin-sensitive transpeptidase (DD-transpeptidase)	14.74
Rv0092	ctpA	Cation transporter P-type ATPase a CtpA	15.00
Rv0202c	mmpL11	Probable conserved transmembrane transport protein MmpL11	14.74
Rv0338c	-	Probable iron-sulfur-binding reductase	24.33
Rv0496	-	hypothetical protein	11.50
Rv0758	phoR	Possible two component system response sensor kinase membrane associated PhoR	18.00
Rv0845	-	Possible two component sensor kinase	11.67
Rv0862c	-	hypothetical protein	12.22
Rv0982	mprB	Two component sensor kinase MprB	16.67
Rv1028c	kdpD	Probable sensor protein KdpD	13.33
Rv1179c	-	hypothetical protein	12.57
Rv1183	mmpL10	Probable conserved transmembrane transport protein MmpL10	10.00
Rv1215c	-	hypothetical protein	12.11
Rv1265	-	hypothetical protein	11.60
Rv1279	-	Probable dehydrogenase FAD flavoprotein GMC oxidoreductase	15.00
Rv1281c	oppD	Probable oligopeptide-transport ATP-binding protein ABC transporter OppD	12.11
Rv1640c	lysX	Lysyl-tRNA synthetase 2 LysX	16.25
Rv1743	pknE	Probable transmembrane serine/threonine-protein kinase E PknE (protein kinase E) (STPK E)	10.33
Rv1746	pknF	Anchored-membrane serine/threonine-protein kinase PknF (protein kinase F) (STPK F)	10.00
Rv1747	-	Probable conserved transmembrane ATP-binding protein ABC transporter	24.83
Rv1783	eccC5	ESX conserved component EccC5 ESX-5 type VII secretion system protein	11.40
Rv1797	eccE5	ESX conserved component EccE5 ESX-5 type VII secretion system protein Probable membrane protein	14.33
Rv1842c	-	hypothetical protein	13.89
Rv2051c	ppm1	Polyprenol-monophosphomannose synthase Ppm1	11.00

Rv2088	pknJ	Transmembrane serine/threonine-protein kinase J PknJ (protein kinase J) (STPK J)	11.58
Rv2460c	clpP2	Probable ATP-dependent CLP protease proteolytic subunit 2 ClpP2 (endopeptidase CLP 2)	10.53
Rv2703	sigA	RNA polymerase sigma factor SigA (sigma-A)	10.00
Rv2933	ppsC	Phenolpthiocerol synthesis type-I polyketide synthase PpsC	13.89
Rv2942	mmpL7	Conserved transmembrane transport protein MmpL7	10.00
Rv2962c	-	Possible glycosyl transferase	10.56
Rv3029c	fixA	Probable electron transfer flavoprotein (beta-subunit) FixA (beta-ETF) (electron transfer flavoprotein small subunit) (ETFSS)	11.60
Rv3245c	mtrB	Two component sensory transduction histidine kinase MtrB	17.37
Rv3365c	-	hypothetical protein	32.00
Rv3519	-	hypothetical protein	12.22
Rv3554	fdxB	Possible electron transfer protein FdxB	12.78
Rv3610c	ftsH	Membrane-bound protease FtsH (cell division protein)	12.40
Rv3627c	-	hypothetical protein	10.00
Rv3693	-	Possible conserved membrane protein	10.56
Rv3764c	tcrY	Possible two component sensor kinase TcrY	16.11
Rv3775	lipE	Probable lipase LipE	21.58
Rv3779	-	Probable conserved transmembrane protein alanine and leucine rich	10.00
Rv3794	embA	Integral membrane indolylacetylinositol arabinosyltransferase EmbA (arabinosylindolylacetylinositol synthase)	10.00
Rv3808c	glfT2	Bifunctional UDP-galactofuranosyl transferase GlfT2	11.67
Rv3869	eccB1	ESX conserved component EccB1 ESX-1 type VII secretion system protein Possible membrane protein	10.00
Rv3876	espI	ESX-1 secretion-associated protein EspI Conserved proline and alanine rich protein	15.56
Rv3885c	eccE2	ESX conserved component EccE2 ESX-2 type VII secretion system protein Possible membrane protein	11.03
Rv3894c	eccC2	ESX conserved component EccC2 ESX-2 type VII secretion system protein Possible membrane protein	42.11
Rv3895c	eccB2	ESX conserved component EccB2 ESX-2 type VII secretion system protein Probable membrane protein	11.67

Supplementary Table 3. Strains used in this work.

Strain	Reference
Mtb H37Rv	Gift from C. Sassetti, University of Massachusetts.
Mtb etfD-tetOFF	This work
Mtb Δ*etfD*	This work
Mtb Δ*etfD* complemented	This work
Mtb Δ*etfD*::*pox3*	This work
Mtb Δ*etfBA*	This work
Mtb Δ*etfBA* complemented	This work
Mtb Δ*etfBA*::*pox3*	This work
Mtb H37Rv::Flag	[1]
Mtb Δ*etfD*::*etfD*-Flag	This work

Supplementary Table 4. Plasmids used in this work.

Plasmid	Reference
pNit-Rec-ET	[2]
pGMCgS-TetOff-13	[3]
pMCK-phsp60-etfD	This work
pMCK-phsp60-etfBA	This work
pMCK-pTB38-pox3	This work
pMEK-phsp60-etfD-flag	This work
pGMCK-hsp60-Flag	[1]

REFERENCES

1 Wang, R. *et al.* Persistent *Mycobacterium tuberculosis* infection in mice requires PerM for successful cell division. *eLife* **8**, doi:10.7554/eLife.49570 (2019).

2 Murphy, K. C., Papavinasasundaram, K. & Sassetti, C. M. Mycobacterial recombineering. *Methods in molecular biology (Clifton, N.J.)* **1285**, 177-199, doi:10.1007/978-1-4939-2450-9_10 (2015).

3 Klotzsche, M., Ehrt, S. & Schnappinger, D. Improved tetracycline repressors for gene silencing in mycobacteria. *Nucleic acids research* **37**, 1778-1788, doi:10.1093/nar/gkp015 (2009).
