# Supplementary material for: Multiple acyl-CoA dehydrogenase deficiency kills Mycobacterium tuberculosis in vitro and during infection
Source: Nat Commun. 2021 Nov 15;12:6593. doi: 10.1038/s41467-021-26941-1 (PMC8593149; doi:10.1038/s41467-021-26941-1)
Supplement: Supplementary file 3 — Description of Additional Supplementary Files [file 41467_2021_26941_MOESM3_ESM.pdf]

Description of Additional Supplementary Files

File Name: Supplementary Data 1
Description: List of species with at least one protein homologous to EtfD, EtfB and EtfA.
